# Treatment of Fracture of the Lower Limb

**Published:** 1918-08

**Authors:** 


**_Remarks on the Treatment of Fracture of the Lower Limb._**
George C. Sneyd, *Lancet*, April 13, 1918.

A great number of old cases exhibit grave deformity. The treatment resolves itself into dealing with (*a*) sepsis; and (*b*) correction of deformity.

A. *Sepsis.* One is tempted to say that the principal treatment of sepsis by suitable incision for drainage is in some degree overlooked. In some cases one has found various treatments by antiseptics and irrigating fluids unsuccessful, or drainage and extension were lacking.

Extension alone helps drainage by preventing pocketing of pus and by allowing free circulation : an immediate fall of temperature ensues in many cases when sufficient extension has not previously been applied.

Too free removal of comminuted bone is not advisable, it may lead to considerable shortening.

The treatment adopted in an early stage should be that of keeping the sinus or wounds clean, while the limb is splinted in the most suitable position with extension, leaving free access for dressings to be applied with as little disturbance as possible to the fracture.

Although many new forms of treatment have been introduced, fomentations remain one of the most useful remedies against sepsis. They are advantageous in cases of cellulitis.

In these conditions good results follow a very minute dose of autogenous vaccine, 1/2 to 1 million streptococcal, accompanied by an injection of serum. The author has seen great benefit result from the use of sulphur and glycerine. Bipp also appears to lessen reaction after operation, such as sequestrotomy.

B. *Correction of Deformity* :

When sepsis is well established it may be very unwise to interfere with deformity. If the case is a recent one, sepsis would be minimized by extension. Three months should be the absolute minimum. Good results cannot be obtained by any one method. One has at times to rely on operation, at others, on various methods of splinting, depending on the individual case.

Lane's method of replacement by open manipulation with powerful bone-lifters, appeals forcibly by its simplicity. Bone-plating in these cases ensures the accurate apposition of the fragments, which could not be gained by other means. But no septic case should be plated.

Sometimes old cases, where the bone is in a decalcified condition, require two or more plates. It is usual to put these cases up in a Hoefftcke's ambulatory splint.

The author has used a Thomas splint frequently, but has always applied weight extension (14-25 lbs) — some times to the extension strapping and at others to a spat.

The Hoefftcke splint has often been used for fracture of the lower limb. It is telescopic and the upper flanges for counter-extension are adjustable.

Sinclair's glue extension or adhesive plaster can be used for the purpose of extension in either the Thomas or the war splint. But a properly moulded spat will fit any foot, and, if correctly applied, will not cause any pressure-sores in the ankle region.

Lateral deformity can be greatly improved even in old cases by applying lateral tension with rubber tubing.

